# Local Anœsthesia by the Application of an Electric Current

**Published:** 1858-07

**Authors:** 


### EDITORIAL DEPARTMENT.

*Local Anœsthesia by the Application of an Electric Current.*—Many, if not most of the readers of the Journal, have doubtless heard of this novel method of producing local anæsthesia, and its application in the extraction of teeth. It was brought to the notice of the Senior Editor about two months ago, by one of the agents of Mr. Jerome B. Francis, of Philadelphia, the inventor, with a request that he would give it a fair trial. Having consented to do so, he requested the agent to place in the infirmary of the Baltimore College of Dental Surgery one of the small galvanic batteries which had been gotten up for the purpose, and, at the time appointed for the experiment, a number of persons, having decayed and aching teeth, presented themselves for the purpose of undergoing what they had been led to believe would be a painless operation, and in nearly every case their hopes were seemingly realized. Between thirty and forty teeth were extracted by Dr. Arthur, late Professor in the Philadelphia Dental College. The experiment at the College was continued two afternoons; on the second day the Senior Editor was not present, but Dr. Arthur informed him that it was quite as successful as on the first, and with a view of ascertaining how much the imagination might influence the feelings of the patient, he, in the case of a boy, who wished to have two teeth extracted, applied the electric current while removing the first; the lad affirmed that he suffered no pain, but in the removal of the second it was not applied, and the pain occasioned by the operation was so great, that he screamed lustily.

The apparent success of these experiments, induced the Senior Editor to apply the invention of Mr. Francis in his private practice, and the result thus far has certainly been very satisfactory—a large majority of his patients, for whom he has extracted teeth, having assured him that they experienced no pain while undergoing the operation, and, as a general thing, those who did suffer stated that the pain was much less than what they usually suffered under the operation. The anæsthetic effect of the electric current seems to be different in different individuals. When the tooth can be grasped and extracted without the instrument coming in contact with the gum, the operation has been apparently completely successful, but when it is pressed against the surrounding soft tissues, the entire current of the electricity does not pass through the tooth, and we presume it is for this reason, that the operation is not then successful.

How it is that local anæsthesia is thus produced we cannot understand, but that it is, at least, in a large majority of the cases, seems to be well established, and wishing to give our readers all the information which we possess upon the subject, we subjoin the following report of the Philadelphia Franklin Institute, and to which, it will be perceived, are attached the names of two dentists well known to the profession. As the method of applying the electrical current is described in this report, it is not necessary for us to repeat what is there said upon the subject.

---

HALL OF THE FRANKLIN INSTITUTE,
*Philadelphia, April* 8, 1858.

The Sub-Committee on Science and the Arts, constituted by the Franklin Institute of the State of Pennsylvania, for the promotion of the Mechanic Arts, to whom was referred for examination the method of extracting teeth, invented by Mr. Jerome B. Francis, of Philadelphia, Penn.

REPORT.

That the object of this invention is the painless extraction of teeth. The method is simple. It consists in attaching the extracting instrument by means of a flexible conductor to one pole (preferably the negative) of an ordinary electro-magnetic machine, while the patient grasps the metallic handle attached to the other pole. An interrupted electrical current thus traverses the body of the patient and the extracting instrument. The intensity of the current is previously graduated by withdrawing the piston of the coil while the patient grasps both the forceps and handle, until it is just distinctly perceptible, and the circuit through the tooth is not completed until the moment at which extraction is to begin.

In regard to the efficacy of the process, the Committee has the following testimony:

1st. 164 teeth were extracted in the presence of the Committee, a report of which cases is herewith submitted.

2d. Members of the Committee have experimented independently with the apparatus. The report of one member who has had it in use four weeks, and extracted between four and five hundred teeth with it, is also submitted.

The Committee is satisfied, from the observation and experiment of its members, that in a large majority of cases of extraction with this apparatus, *no pain whatever* is felt by the patient.

To test the question whether the effect might not be simply mental, the circuit was broken without the patient being aware of it, when the usual pain was experienced, although, in the same patient, and on the same occasion, teeth had been removed while the current was flowing without causing pain.

In the less successful cases, the teeth were broken and diseased below the level of the gum, and the pain, in adjusting the forceps previous to the completion of the circuit and the extraction, considerable.

The sensation produced by the passage of the current is not painful, it being so adjusted as to be *just perceptible* to the patient. The Committee believes its use to be entirely without danger, and not likely to be followed by any unpleasant after effects.

The whole apparatus is cheap, the operator requires no new instruments, except the battery and coil, and the application demands no more time, and scarcely more trouble than the ordinary method.

In these respects its use contrasts favorably with that of general anæsthetics, which are troublesome and tedious in their application, and not free from danger, or the employment of freezing mixtures to the jaw, for the purpose of producing local anæsthesia; a process which cannot be recommended as either harmless or efficient.

The Committee believes the employment of electricity in the manner and for the object indicated, to be original with Dr. Francis. It is true, that the heat developed by the passage of a considerable current through fine platinum wire, has been employed to cauterize ulcers of the throat, and to destroy the nerves of teeth. There is obviously, however, no parallelism in these cases. The use of the electrical current in neuralgia presents more analogy; in these cases, however, the current is used for a long period, and with the object of inducing a change in the morbid state of the parts, rather than as a means of removing pain at once; whereas, to be effective in extraction, the current must be applied at the moment of extraction; if allowed to flow through the tooth for a very short time before the operation, its effect is diminished or lost.

As to the theory of these very singular and unexpected results, the Committee does not express an opinion; of the fact it is fully satisfied.

In view of the importance of the discovery, the Committee recommends to Mr. Francis, the award of the Scott's medal and premium. All of which is repectfully submitted.

[Signed,] Wm. Hamilton, *Actuary.*

---

Philadelphia, *April* 3*d*, 1858.

*To the Sub-Committee of Science and the Arts, of Franklin Institute, on Francis' Invention:*

Gentlemen:

I have deemed it my duty to submit to you the following statement, for fear I might not be able to attend at your meeting.

When I first heard of Mr. Francis' Invention, my impression of it was very unfavorable; more, perhaps, for the reason that I did not understand how the electricity was applied. Having heard from several of my patients that teeth had actually been extracted without pain, I felt it my duty to try some experiments with it. The first experiment I tried was on three teeth of my son's. I attached one of the poles of the electro-magnetic machine to the forceps, and fitted on the tooth. I then had my son grasp firmly a handle attached to the other pole of the machine, and at the same time I drew the tooth.

The rod of the electro-magnetic machine had been previously so adjusted, that when my son (the patient) grasped the handle in one hand, and the forceps in the other, he could only feel a slight and pleasant sensation of electricity. The experiment was entirely successful, the tooth was extracted (my son says) without pain. I was just as successful with the other two, and also on some teeth which I extracted from my servant girl. I have, up to this time, extracted with this process between four and five hundred teeth, and have been successful with ninety-five per cent. of them.

This process has, of course, entirely changed my first impression of the invention. I have now the most exalted opinion of it, and would not extract teeth without its aid upon any account.

When a patient has more than one tooth to be extracted, I find it saves me much time, for, by the old way, when I had extracted one tooth, I found it very difficult to persuade the patient to have another pulled, which sometimes consumed a great deal of time. By this process, I can go on pulling, as witnessed in the case recorded in my book as No. 3, in which four stumps were extracted in forty (40) seconds, and in No. 4, four teeth were extracted in fifteen (15) seconds, and waited only fifteen seconds, and then extracted two more in ten seconds. Case No. 6, had five teeth pulled in 30 seconds. Case No. 11, had five teeth extracted in 35 seconds, after an interval of thirty seconds, had six in thirty seconds more, and after an interval of thirty seconds, had two more in eight seconds, and after an interval of two and a half minutes, had one more incisor, making 14 in all.

I also found, contrary to my first impression, that teeth diseased at the roots could be extracted with but slight pain, and sometimes without any, and without any disagreeable feeling from the current, as witnessed in case No. 25; a lady, twenty-five years of age, had three teeth taken out, one of them very badly diseased at the root. She exclaimed, Oh! that don't hurt! and so with several other cases. There is sometimes an after-pain in extracting diseased teeth. It occurs immediately after the tooth leaves the socket; it is probably caused by the cold air coming in contact with the diseased part thus exposed.

Teeth extracting is the most unpleasant part of dentistry, and I am happy to state that, by my use of this process, I have been saved much of the unpleasantness occasioned by giving my patients so much pain. A young man had a tooth extracted by me yesterday, he said he had always a great deal of difficulty in having his teeth extracted, but with this one, he said he felt no pain. He was quite delighted, and thanked me; something, he said, he had never done to a dentist before.

Numerous instances of the same kind have occurred in my use of this invention. I have found that the hemorrhage is in no degree increased by this process, and I have heard of no person who experienced any subsequent pain or inconvenience. In no case have I found the use of this invention in any way objectionable.

Very respectfully, yours, &c.

W. S. WILKINSON.

B. Howard Rand, M. D.
Franklin Peale, Esq.
J. Aitken Meigs, M. D.

---

The report of the Committee on file in the Franklin Institute is signed by

| | |
|---|---|
| B. Howard Rand, M. D. | Franklin Peale, |
| W. H. Hazard, M. D. | J. Aitken Meigs, M. D. |
| Henry Hartshorne, M. D. | J. De Haven White, M. D. |
| Wm. S. Wilkinson, | Edward Townsend, D. D. S. |

---

The exclusive right to the application of electricity for the production of local anæsthesia in the extraction of teeth, has been secured to the inventor, Mr. Francis, as will be seen by the following schedule and letters patent: EDS.

---

*The Schedule referred to in these Letters Patent, and making parts of the same.*

TO ALL WHOM IT MAY CONCERN:

Be it known, that I, Jerome B. Francis, of the City of Philadelphia, and State of Pennsylvania, have invented a new and useful improvement in instruments for removing teeth; and I do hereby declare the following to be a full and exact description of the same, reference being had to the annexed drawing, making part of this specification, which represents a perspective view of my improvement.

My improvement consists in combining with a common dental forceps an electro-magnetic machine, in such manner that a wire from a negative pole of the machine shall form a metallic connection with that part of the forceps that grasps the tooth, and that the positive pole of the machine shall be connected with the patient's hand by a metallic connection. The handles of the forceps, which are held by the operator, are better to be insulated with gutta-percha, or similar non-conducting substance.

In the accompanying figure, A represents an ordinary electro-magnetic machine, with the battery, B, attached. D represents the ordinary dental forceps. A wire, C passes from the negative pole, P, of the electro-magnetic machine to the point, *d*, of the forceps, where a close metallic connection is made. On the inner side of the forceps, at the point *d*, a small metallic cup is placed, and a small copper stem projects from the opposite side, *e*, of the forceps. As the parts *f* and *g* of the forceps close upon the tooth where it is surrounded by the gum, the induced current from the electro-magnetic machine passes through the wire C, and across from *d* to *e*, and thus applies itself around the whole tooth in the vicinity of the nerves, and so affects also the nerves, rendering them temporarily insensible. The patient, or person operated on must hold in one of his hands the extremity of the other wire, H, so as to complete the circuit through his body. I represents the hands of the operator grasping the forceps, K the hand of the patient grasping the wire passing to the positive pole of the machine. The electro-magnetic machine has a sliding rod, by which the induced current may be varied in intensity. The intensity of the current to be passed through the patient's tooth should be graduated, by observing in advance how much he can conveniently bear when he grasps the extremity of the wires H and C in each hand. A lttle practice will enable the dentist to determine this readily. The electro-magnetic machine, A, is of the ordinary form employed for medical purposes, and consists of a battery of one cell, a primary coil and inducing coil, a small electro-magnet for breaking and closing the circuit through the wires C and H and the patient's body. Any other form of electro-magnetic machine may be employed.

I have described above, particularly, the machine frequently called an electro-magnetic machine, and which is used for medical purposes. There are several other forms of machine, sometimes classified as electro-magnetic, or magneto-electric, which are known to be equivalent in their action on the nervous system to the one above described—such for example, as the double helices, or coils composed of two coils of wire, one surrounding the other, one being a quantity helix, or coil, and connected with the battery, and the other an intensity helix or coil, and to be connected with the body of the patient.

Instead of using a little electro-magnet break-circuit in the first helix as shown in the drawing, a clock-work break-circuit, or electrotome, may be used, or a rasp may be used, in connection with the aid of an assistant, for breaking and closing the circuit; so also there are several forms of magneto-electric machines in which permanent magnets are used to induce, by mechanical action, a magneto-electric current in a coil surrounding a revolving soft iron armature. In all these cases the same peculiar effect on the nerve of the patient's tooth would result if either of these machines were combined with the forceps, inasmuch as they are all well known and to be equivalents in applying electricity to the body for medical purposes. A direct circuit from the battery might also be combined with the forceps, and either with or without the aid of an interposed break-circuit the same effect would take place to a great degree, although the use of such a battery, of the proper intensity, would probably be found much more inconvenient than the magneto-electric (or as they are sometimes called, electro-magnetic,) machines above named.

Instead of a metallic conductor, to connect the electro-magnetic machine with the forceps, any other kind of conductor might be used—or the operator's hands might be used for one conductor. I prefer to connect the negative pole of the machine with the forceps; but this is not indispensable, as the positive pole will also answer. The little pieces, *f* and *g*, are not indispensable, because the forceps itself conveys the current very rapidly to the tooth, around the whole body of the tooth.

Having thus described my improvment, what I claim as my invention, and desire to secure by Letters Patent, is the combination of the electro-magnetic machine, or its equivalent, with the dental forceps, for removing teeth without pain; arranged and operating substantially in the manner above described. J. B. FRANCIS.

*Witnesses,* { J. H. B. JENKINS, CHARLES D. FREEMAN.

---

ISSUE No. 20,390.

TO ALL WHOM THESE LETTERS PATENT SHALL COME:

*Whereas,* Jerome B. Francis, of Philadelphia, Pa., has alleged that he has invented a new and useful improvement in the method of extracting teeth, (he having assigned his right, title and interest in said improvement to William Harper, Jr., of said Philadelphia, and said Harper having re-assigned the same to said Jerome B. Francis, and the said Jerome B. Francis having assigned the same to James J. Clark, of said Philadelphia,) which he states has not been known or used before his application; has made oath that he is a citizen of the United States; that he does verily believe that he is the original and first inventor or discoverer of the said improvement, and that the same hath not, to the best of his knowledge and belief, been previously known or used; has paid into the treasury of the United States, the sum of thirty dollars, and presented a petition to the Commissioner of Patents, signifying a desire of obtaining an exclusive property in the said improvement, and praying that a Patent may be granted for that purpose.

These are, therefore, to grant, according to law, to the said James J. Clark, his heirs, administrators, or assigns, for the term of fourteen years from the twenty-fifth day of May, one thousand eight hundred and fifty-eight, the full and exclusive right and liberty of making, constructing, using, and vending to others to be used, the said improvement, a description whereof is given in the words of the said Jerome B. Francis, in the schedule hereunto annexed, and is made a part of these presents.

IN TESTIMONY WHEREOF, I have caused these letters to be made patent, and the seal of the Patent Office has been hereunto affixed. Given under my hand, at the City of Washington, this twenty-fifth day of May, in the year of our Lord one thousand eight hundred and fifty-eight, and of the independence of the United States of America, the eighty-second.

JACOB THOMPSON, *Secretary of the Interior.*

*Countersigned and sealed with the seal of the Patent Office.*

J. HOLT, *Commissioner of Patents.*

